# Genetic Diversity in the Major Capsid L1 Protein of HPV-16 and HPV-18 in the Netherlands

**DOI:** 10.1371/journal.pone.0152782

**Published:** 2016-04-12

**Authors:** Audrey J. King, Jan A. Sonsma, Henrike J. Vriend, Marianne A. B. van der Sande, Mariet C. Feltkamp, Hein J. Boot, Marion P. G. Koopmans, on behalf of Medical Microbiological Laboratories and Municipal Health Services

**Affiliations:** 1 Centre for Infectious Disease Control, National Institute for Public Health and the Environment (RIVM), Bilthoven, The Netherlands; 2 Julius Centre for Primary Care and Public Health, University Medical Centre, Utrecht, The Netherlands; 3 Department of Medical Microbiology, Leiden University Medical Center, Leiden, The Netherlands; Albert Einstein College of Medicine, UNITED STATES

## Abstract

**Objectives:**

Intratypic molecular variants of human papillomavirus (HPV) type-16 and -18 exist. In the Netherlands, a bivalent vaccine, composed of recombinant L1 proteins from HPV-16 and -18, is used to prevent cervical cancer since 2009. Long-term vaccination could lead to changes in HPV-16 and -18 virus population, thereby hampering vaccination strategies. We determined the genetic diversity of the L1 gene in HPV-16 and -18 viral strains circulating in the Netherlands at the start of vaccination in order to understand the baseline genetic diversity in the Dutch population.

**Methods:**

DNA sequences of the L1 gene were determined in HPV-16 (n = 241) and HPV-18 (n = 108) positive anogenital samples collected in 2009 and 2011 among Dutch 16- to 24-year old female and male attendees of the sexually transmitted infection (STI) clinics. Phylogenetic analysis was performed and sequences were compared to reference sequences HPV-16 (AF536179) and HPV-18 (X05015) using BioNumerics 7.1.

**Results:**

For HPV-16, ninety-five single nucleotide polymorphism (SNPs) were identified, twenty–seven (28%) were non-synonymous variations. For HPV-18, seventy-one SNPs were identified, twenty-nine (41%) were non-synonymous. The majority of the non-silent variations were located in sequences encoding alpha helix, beta sheet or surface loops, in particular in the immunodominant FG loop, and may influence the protein secondary structure and immune recognition.

**Conclusions:**

This study provides unique pre-vaccination/baseline data on the genetic L1 diversity of HPV-16 and -18 viruses circulating in the Netherlands among adolescents and young adults.

## Introduction

Human papillomaviruses (HPVs) are small double stranded DNA viruses that have the potential to infect mucosal and cutaneous human epithelial cells. Over 160 HPV types have been identified based on the DNA sequence of the L1 open reading frame of which about 60 types infect the human anogenital tract [1] and are sexually transmitted. A persistent infection with any of at least 12 high-risk HPV types is associated with the development of human cervical cancer [2]. Of these high-risk types, HPV-16 and HPV-18 are responsible for about 70% of cervical cancers [3].

Naturally occurring intratypic molecular variants of HPV-16 and -18 are known to occur and have been shown to be specific or more prevalent in certain parts of the world [4]. Intratypic molecular variants of HPV-16 and -18 are characterized as isolates of the same HPV type that differ in the nucleotide sequence of L1 for less than 10%. Despite phylogenetic relatedness HPV intratypic variants can differ in pathogenicity and in oncogenic potential [5–10]. For HPV-16 phylogenetic analyses revealed 4 major intratypic variant lineages:1. European (E/A), 2. Asian-American (AA/D), 3. African-1 (Af-1/B) and 4. African-2 (Af-2/C)[1]. These variants have been shown to differ in persistence and progression to cancer. Intratypic variants for HPV-18 are also described but the evidence for differences in persistence or progression to (pre)cancer is less clear [1].

HPV vaccination of sexually naïve girls has been introduced in many countries in order to prevent cervical cancers caused by persistent infection with HPV-16 or -18. To what extent HPV vaccination will affect the distribution of HPV types and variants in a (partially) vaccinated population is not yet known. Therefore, monitoring of type-specific HPV prevalence before and after the start of HPV vaccination is of great importance. Previous HPV monitoring studies have shown that the three most prevalent high-risk HPV types (among women) at the introduction of HPV vaccination in the Netherlands were HPV-16, HPV-51 and HPV-52 [11–13].

Since 2009, a bivalent vaccine, containing virus-like particles (VLPs) composed of pentamers of recombinant major capsid protein L1 from HPV-16 and -18, is used for vaccination of young girls in the Netherlands. This HPV vaccine is known to elicit high-titre neutralizing antibodies directed against the L1 protein and confer type-specific and long-lasting protection against a persistent infection and cervical abnormalities caused by HPV-16 and -18 [14]. An additional protective vaccine efficacy against infections associated with HPV-31, -33 and -45 have been shown [15]. On the surface of the pentamers, specific loop structures of the L1 protein containing hypervariable immunodominant regions are exposed [16]. Polymorphism within these loops is likely to result in the generation of neutralizing antibodies of different binding affinities [17]. At present, it is not known if immunity elicited against one HPV-16 or -18 variant can protect against infection from another variant with equal efficiency. Long-term vaccination with one HPV-16 or -18 variant of the L1 proteins could therefore lead to changes in HPV-16 and -18 virus population induced by selective pressures.

In order to detect a long-term effect of L1 vaccination on the HPV-16 and/or -18 virus population, knowledge of the genetic diversity of L1 gene in HPV-16 and -18 viruses circulating in the Netherlands at the start of vaccination is required. The aim of this study was to determine the genetic diversity in the L1 protein and HPV variant distribution of circulating HPV-16 and -18 viral isolates found in young adolescents before the start of HPV-16/-18 vaccination, at baseline.

## Material and Methods

### Study population and design

Between February and April 2009, a cross-sectional study was started in 12 sexually transmitted infection (STI) clinics spread throughout the Netherlands prior to the introduction of vaccination. Follow-up surveys in this setting have been conducted every two years since 2009. For the results presented here only samples obtained in 2009 and 2011 were analyzed. The study population and methods have been described in detail previously [12, 13]. The study was approved by the Medical Ethical Committee of the University of Utrecht, the Netherlands. This committee has confirmed in writing that they have waived the need for separate ethical approval and the need for written consent.

### Clinical specimens, HPV DNA detection and Sample selection

Samples were obtained from anogenital (i.e.vaginal/penile/anal) swabs collected in 2009 and the follow up year 2011 within the PASSYON (PApillomavirus Surveillance among Sti clinic YOungsters Netherlands) study among Dutch 16- to 24-year old male and female attendees of STI clinics. Details of the PASSYON study were described elsewhere [12, 13]. All swabs were suspended in 1 ml universal transport medium buffer and stored at -20°C until processing. After thawing, swabs were vortexed and 200 μl of the sample was spiked with phocine herpes virus-1. DNA was subsequently extracted using the MagnaPure platform (Total Nucleic Acid Isolation Kit, Roche) and eluted in 100 μl elution buffer. HPV-DNA was amplified using the SPF10 primer set according to the manufacturer’s instructions (DDL Diagnostic Laboratory, the Netherlands). HPV-specific amplicons were detected using the DNA enzyme-linked immunoassay (HPV-DEIA, DDL Diagnostic Laboratory, the Netherlands). Amplicons of HPV-positive samples were subsequently analyzed in the Line probe assay (HPV-LiPA, DDL Diagnostic Laboratory, the Netherlands) in order to determine the specific HPV type present in the sample. HPV isolates for sequencing were selected from a large set of samples previously typed for HPV based on successful L1 gene PCR results. These samples were analyzed further, in order to determine the genetic diversity in the L1 gene.

### PCR sequencing

HPV-16 (n = 241) and HPV-18 (n = 108) positive samples were selected for further molecular characterization of the L1 gene (1596 bp for HPV-16 and 1707 bp for HPV-18) by sequence analysis. The entire L1 gene of HPV-16 and HPV-18 was amplified in three overlapping fragments using the primers listed in S1 Table [18]. The GenBank reference sequences used for primer design for HPV-16 were (AF536179, AF402678, AF536180, AF472509, K02718, FJ006723) and for HPV-18 (X05015, EF202147, EF202152). PCR amplification of the fragments was performed in 50 μl containing 3 μl extracted DNA, 1.25 Units Amplitaq Gold (Applied Biosystems), PCR II buffer (Applied Biosystems), 2 mM MgCl_2_, 0.2 mM dNTPs (Roche) and 10 pmol of each primer. The cycling conditions were as follows: 15 min 95°C denaturation; 15 sec 95°C, 30 sec 55°C, 90 sec 72°C for 35 cycles; 10 min 72°C final extension. Amplicons were visualized on 2.2% agarose gels. PCR products were treated with ExoSAP-IT® and sequenced by automated DNA sequencing using Big Dye Terminator 3.1.

### Molecular characterization and phylogenetic analysis

All sequences from a given sample were combined and used to construct a contig. Contig sequences were aligned using Bionumerics version 7.1 and compared with HPV-16 European German reference sequence AF536179 (sublineage A2), or HPV-18 reference sequence X05015 (lineage A/European). For clustering of HPV-16 the L1 gene, GenBank reference sequences from K02718 = NC001526 (sublineage A1/European), HQ644236 (sublineage A3/ European), AF534061 (sublineage A4/European-Asian), AF536180 (sublineage B1/African-1a), HQ644298 (sublineage B2/African-1b) and AF472509 (sublineage C, African-2), HQ644257 (sublineage D1-North American), AY686579-(sublineage D2, Asian-American), AF402678 (sublineage D3, Asian-american) and AF043286 (vaccine variant) were included in the analyses. For clustering of HPV-18 the L1 gene, GenBank reference sequences from X05015 (lineage A/European), AY262282 (sublineage A1, European-1), EF202146 (sublineage A2, European-1), EF202151 (sublineage A4, European-2), GQ180787 (sublineage A5/ European) were used. HPV-16 and -18 L1 sequences sequenced here were deposited into the NCBI GenBank database (accession numbers KU70477-KU707717 for HPV-16 L1 sequences and KU707718-KU707825 for HPV-18 L1 sequences). The presence of the variations found infrequently, in one, two or three strains were confirmed by resequencing at least 2 additional times.

To estimate selection pressure acting on HPV-16 and HPV-18 L1 sequences, codon-specific, non-synonymous (dN) and synomymous (dS) substitutions rates were inferred by using Nei- Gojobori method [19] and Jukes- Cantor Model with the program SNAP v2.11 (http://www.hiv.lanl.gov) [20]. Non-synonymous (dN) and synomymous (dS) substitutions rates were calculated and compared, the ratio of dN/dS was calculated.

## Results

### HPV-16 L1 genetic diversity

**Fig 1:**
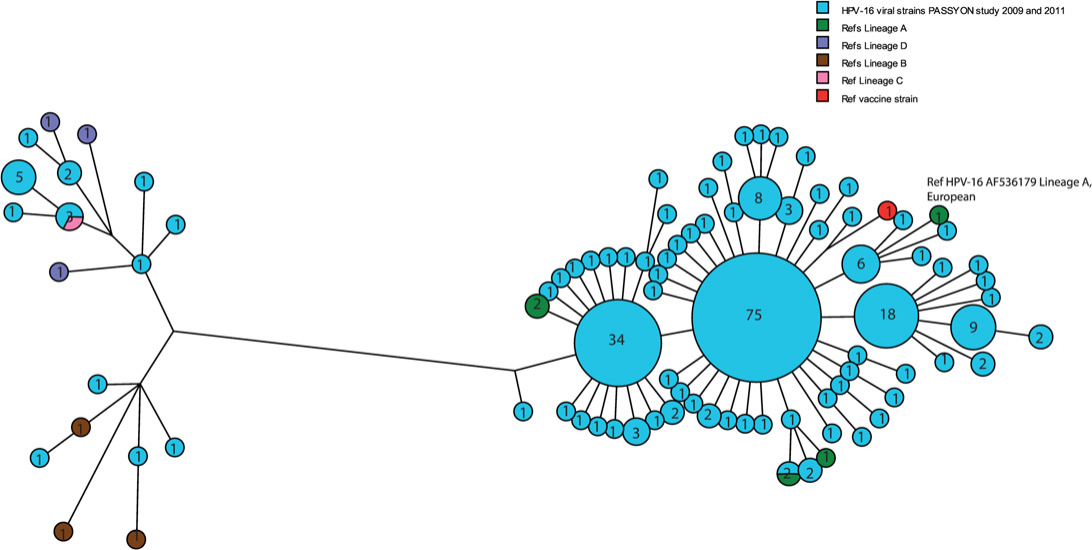
Maximum parsimony tree based on nucleotide sequences of L1 HPV-16 gene. Study sequences of 241 HPV-16 positive samples collected in 2009 and 2011 are shown in blue. Reference sequences of European/A lineages (GenBank reference AF536179 K02718 = NC001526, HQ644236, AF534061) are shown in green, African/B lineages (GenBank references AF472508, HQ644298 and AF536180) in brown. African-2/C lineage GenBank reference AF472509 is shown in pink. References sequences from lineage D are shown in purple HQ644257 (sublineage D1-North American), AY686579-(sublineage D2, Asian-American) AF402678 (sublineage D3, Asian-American). HPV-16 variant used in the vaccine (GenBank reference AF043286) is shown in red. The number given in each circle indicates the number of viral strains with the same sequence.

L1 HPV-16 sequences were determined and analyzed by aligning the entire L1 gene from 241 HPV-16 positive samples collected in the PASSYON study rounds 1 (2009) and 2 (2011) [13]. Forty-six percent (46%) of all HPV-16 positive samples in these two rounds (Table 1), which are considered as pre-vaccination samples, were sequenced in the present study. The characteristics of the total study population and the strains selected for sequencing are shown in Table 1. Phylogenetic analysis of all L1 sequences clustered the HPV-16 variants in two groups. The majority of the HPV-16 strains 223/241 (93%) had high similarity to the HPV-16 European reference (GenBank reference AF536179, lineage E/A) and other lineage A reference strains (Fig 1). A small subset 18/241 (7%) clustered with the African types (GenBank references AF472508 (Af-1/B), AF472509 (Af-2/C)) and Asian-American type D3 (AF402678). Non-European HPV-16 variants were collected from persons who self-reported to belong to the Surinam or Antillean population group, Dutch population and from unknown population group respectively, in 11%, 50% and 33% of 18 non-European variants (not shown). The most common lineage E/A variant was detected in 31% (75/241, Fig 1) of the samples and differed from the HPV-16 European reference (GenBank reference AF536179) by 2 silent variations and from the HPV-16 vaccine strain (GenBank reference AF043286) by 3 silent variations.

**Table 1:** Characteristics of study population.

	HPV-16 positive sequenced		HPV-18 positive sequenced	
	N = 241 of 524		N = 108 of 311	
	n seq/n total	*%*	n seq/n total	%
**Year of participation**				
2009	97/249	*40*	42/127	*39*
2011	144/275	*60*	66/184	*61*
**Age**				
16–18	22/45	*9*	5/17	*5*
19–21	92/203	*38*	47/125	*43*
22–24	127/276	*53*	56/169	*52*
**Gender**				
Female	214/421	*89*	87/218	*81*
Male	27*/103	*11*	21*/93	*19*
**Population group**				
Dutch	207/444	*85*	92/269	*85*
Non-Dutch	27/73	*10*	15/41	*14*
unknown	7/7	5	1/1	*1*

Differences in sequenced (= n seq) and total group (= n total) were tested with Chi- square test, all were non-significantly different except for gender.

* Indicates significant difference between the number of HPV-16 and -18 sequenced samples from males compared to male present in the total group (P<0.05). The number of HPV-16 and -18 sequenced samples from males was lower.

In total we identified ninety-five single nucleotide polymorphisms (SNPs) among the observed Dutch HPV-16 strains compared to the reference strain AF536179. Of these SNPs, 68/95 (72%) were synonymous variations and 27/95 (28%) were non-synonymous variations. The majority of the non-synonymous variations 21/27 (78%) was located in the L1 region encoding alpha helix, beta sheets, surface loops or connecting loops, in particular in the immunodominant FG loop, and therefore may influence the protein secondary structure. The position of the non-synonymous variations is shown in Table 2. Twelve of these non-synonymous variations were located in structured areas of the L1 protein and were, to our knowledge, not described previously. Ten of these twelve variations were identified each in one strain only, eight were found in strains similar to European strains and two in those more similar to the non-European lineages. Resequencing confirmed the presence of the variations found infrequently (in one, two or three strains) as was indicated in Table 2.

**Table 2:** Non-synonymous nucleotide sequence variations of HPV-16 L1.

Nucleotide position from start Ref	5562	5629	5862	5920	6018	6163	6166	6178	6216	6432	6443	6445	6445	6480
AF536179	C	T	C	C	G	C	A	A	G	G	A	A	A	T
**Variation-frequency (n)**	18	2*	19	1*	1*	18	1*	7	2*	54	1*	1*	1*	8
**Variation-frequency (%)**	7.5	0.8	7.9	0.4	0.4	7.5	0.4	2.9	0.4	22.4	0.4	0.4	0.4	3.3
**Change to nucleotide**	G	C	T	G	A	A	C	C	A	A	C	C	G	C
**Secondary structure**		** **	**β-C**	**β-D**	**DE-loop**	**EF-loop**	**EF-loop**	**EF-loop**	**EF-loop**	**FG-loop**	**FG-loop**	**FG-loop**	**FG-loop**	**FG-loop**
**NUCL POS from start L1 ML-W**	-74	-7	226	284	382	527	530	542	580	796	807	809	809	844
**AA pos L1 from M-LW**	-24	-2	76	95	128	176	177	181	194	266	269	270	270	282
**AA TRIPLET**	CAG	TTT	CAT	ACA	GAC	ACC	AAT	AAT	GTT	GCT	GAA	AAT	AAT	TCT
**AA SWITCH**			H>Y76	T>R95	D>N128	T>N176	N>T177	N>T181	V>I194	A>T266	E>D269	N>T270	N>S270	S>P282
												AA-Coding region		

6489	6490	6505	6562	6693	6801	6822	6940	7058	7110	7125	7138	7139
A	A	A	A	A	A	T	C	G	T	A	A	A
1*	1*	1*	1*	15	3*	3*	1*	18	1*	1*	1*	2*
0.4	0.4	0.4	0.4	6.2	1.2	1.2	0.4	7.5	0.4	0.4	0.4	0.8
C	C	G	G	C	T	C	T	T	C	G	C	C
**FG-loop**	**FG-loop**	**FG-loop**	**β-G2**	**HI-loop**	**α-2**	**α-3**	**connecting loop**	**α-5**	** **	** **	** **	** **
853	854	869	926	1057	1165	1186	1304	1422	1474	1489	1502	1503
285	285	290	309	353	389	396	435	474	492	497	501	501
AAT	AAT	AAT	AAA	ACT	ACA	TCC	GCA	TTG	TCA	ACT	AAA	AAA
N>H285	N>T285	N>S290	K>R309	T>P353	T>S389	S>P396	A>V435	L>F474	S>P492	T>A497	K>T501	K>N501
												

Nucleotide positions of the reference sequence AF536179 are shown on the top of the figure. The sequence of the reference is shown below the positions. Variation frequencies are shown. Secondary structured areas and connecting loops are indicated. Variations in boxed areas were newly found in this study. The nucleotide position from start of L1 and the AA position of L1 is given below the secondary structure. The AA-triplet sequence encoding the amino acid sequence in the reference is shown. Amino acid switches are shown at the bottom of the figure.

* Indicates variation frequencies of 3 or less, these variations/changes were confirmed by resequencing.

We calculated the non-synonymous/synonymous substitution rates for HPV-16 L1 amino acid residues. Based on the HPV-16 sequences analyzed here the dN/dS ratio was 0.176 suggesting that for the entire L1 protein no evidence for positive selection was found (P<0.01).

### HPV-18 L1 genetic diversity

**Fig 2:**
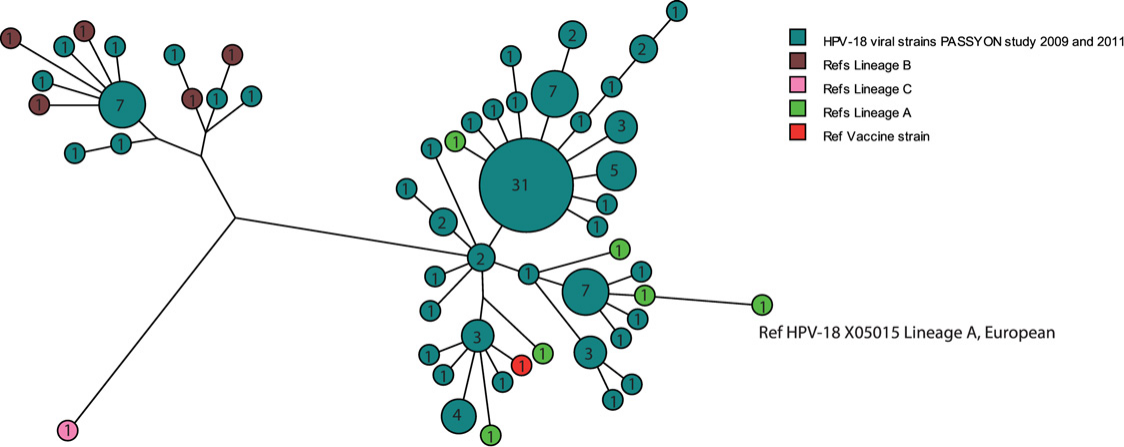
Maximum parsimony tree based on nucleotide sequences of L1 HPV-18 gene. Study sequences of 108 HPV-18 positive samples collected in 2009 and 2011 are shown in blue. Reference sequences of European/A lineages (GenBank reference X05015, AY262282 (sublineage A1), EF202146 (sublineage A2), EF202147 (sublineage A3), EF202151 (sublineage A4), CQ180787 (sublineage A5)), African/B lineages (GenBank references EF202153, EF202154, EF202155 (sublineage B1)) KC470225 (sublineage B2) EF202152 (sublineage B3), and reference for lineage C (KC47229) are shown in green, brown and pink respectively. HPV-18 variant used in the vaccine (GenBank reference AY863161) is shown in red. The number given in each circle indicates the number of viral strains with the same sequence.

L1 HPV-18 sequences were determined and analyzed by aligning the L1 contigs for in total 108 HPV-18 positive samples from the PASSYON study, which is 35% of the HPV-18 positive samples (Table 1). Table 1 shows the characteristics of the total study population and the strains selected for sequencing. Phylogenetic analysis of all L1 sequences showed that the majority 93/108 (86%) of the HPV-18 viral strains have similarity to the HPV-18 European reference (GenBank reference X05015 lineage E/A) (Fig 2). A smaller subset (15/108 (14%)) clustered with the African types (GenBank references EF202154, EF202153 lineage Af/B). Fifteen non-European variants were found, 73% of these were isolated in persons with self-reported non-Dutch origin mostly with a self-reported Surinam or Antillean origin, 27% persons reported to be Dutch and for 0.3% the origin was unknown (not shown). The most common L1 sequence type was detected in 31/108 (29%) of the samples and differed from the HPV-18 European reference (GenBank reference X05015) by 8 (2 silent and 6 non-silent) variations and by 4 (1 silent and 3 non-silent) variations from the vaccine strain (GenBank reference AY863161).

Seventy-one (71) single nucleotide polymorphism (SNPs) were identified among the 108 Dutch HPV-18 strains related to the reference strain X05015. Forty-two of seventy-one (42/71) (59%) were synonymous variations and 29/71 (41%) were non-synonymous variations. For HPV-18 fourteen of the non-synonymous variations 14/29 (48%) were located in sequences encoding the alpha helix, beta sheet, surface loops or connecting loops. The position of the non-synonymous variations is shown in Table 3. To our knowledge only 6 of the 29 non-synonymous variations were described before. Novel variations were mostly found in multiple viral strains (Table 3) and both in strains similar to European and non-European lineages. All variations found in one, two or three strains were confirmed by resequencing.

**Table 3:** Non-synonymous nucleotide sequence variations of HPV-18 L1.

Nucleotide position from start Ref	5458	5467	5496	5497	5503	5505	5521	5560	5563	5573	5581	5584	5619	5701
X05015	T	A	C	A	G	C	G	A	A	A	G	A	T	C
**Variation frequency (n)**	3*	1*	1*	6	107	10	1*	1*	2*	9	17	15	59	108
**Variation frequency (%)**	2.8	0.9	0.9	5.5	99.1	9.2	0.9	0.9	1.8	8.3	15.7	13.9	54.6	100
**Change to nucleotide**	C	C	T	C	A	A	A	G	G	C	A	G	A	G
**Secondary structure**	** **	** **	** **	** **	** **	** **	** **	** **	** **	** **	** **	** **	** **	**β-B1**
**NUCL POS start from L1 at M-LW**	-154	-145	-116	-115	-109	-107	-91	-52	-49	-39	-31	-28	7	89
**AA pos L1 from M-LW**	-52	-49	-39	-39	-37	-36	-31	-18	-17	-14	-11	-10	3	30
**AA TRIPLET**	TTA	CAT	CAC	CAC	CGG	CCC	AGT	CAT	TAT	TTA	AGA	AAC	TTG	CCC
**AA SWITCH**													L> M3	P>R30
														

5775	5875	5920	6430	6441	6460	6579	6581	6625	6626	6921	6924	6925	6949	7086
G	C	C	A	A	C	G	T	C	C	G	A	A	A	A
1*	98	15	12	1*	108	15	15	108	15	1*	1*	1*	3*	2*
0.9	90.7	13.9	11.1	0.9	100	13.9	13.9	100	13.9	0.9	0.9	0.9	2.8	1.8
A	A	T	C	C	G	A	C	G	T	A	C	C	G	G
**BC loop**	**CD loop**	**β-D**	**FG loop**	**FG loop**	**FG loop**	**G2 -H1 loop**	**G2 -H1 loop**	**H1 H2 loop**	**H1 H2 loop**	**connecting loop**	**connecting loop**	**connecting loop**	** **	** **
163	263	308	818	829	848	967	969	1013	1014	1309	1312	1313	1337	1474
55	88	103	273	277	283	323	323	338	338	437	438	438	446	492
GGT	ACT	GCT	CAA	ATT	CCT	GTT	GTT	CCC	CCC	GAA	AAT	AAT	AAG	ACT
G>S55	T>N88	A>V103	Q>P273	I>L277	P>R283	V>I323	V>I323	P>R338	P>R338	E>K437	N>H438	N>T438	K>R446	T>A492
				AA- Coding region										

Nucleotide positions of the reference sequence X05015 are shown on the top of the figure. The sequence of the reference is shown below the positions. Variation frequencies are shown. Secondary structured areas and connecting loops are indicated. Variations in boxed areas were newly found in this study. The nucleotide position from start of L1 and the AA position of L1 is given below the secondary structure. The AA-triplet sequence encoding the amino acid sequence in the reference is shown. Amino acid switches are shown at the bottom of the figure.

* Indicates variation frequencies of 3 or less, these changes were confirmed by resequencing.

Non-synonymous and synonymous substitution rates for HPV-18 L1 amino acid residues were calculated based on the HPV-18 L1 gene sequences analyzed here and shown be significantly different (P<001). No evidence for positive selection was found for the entire L1 gene since the dN/dS ratio was 0.172 suggesting that it was subject to purifying selection.

## Discussion

Human papillomavirus (HPV) vaccination using VLPs composed of the L1 protein has been widely implemented in many countries, with vaccines targeting high-risk HPV-16 and -18 alone, or additional HPV types. While type-specific protection is expected, genotypic and phenotypic variants within HPV types have been recognized, with some evidence for geographic association of this diversity and differences in progression to cervical intraepithelial neoplasia grade 3 (CIN3). At present it is not known if immunity to viruses within an HPV-type is equally efficient for all variants, with some evidence that this may not be the case: although studies have suggested that immunization with L1 VLPs of an European variant induces antibodies able to neutralize different HPV-16 variants [21]. Other studies have shown evidence for variant specific neutralizing epitopes [22]. Understanding such diversity is important as the selective immunological pressure in a (fully) vaccinated population could lead to selective displacement of variants within the HPV types covered by the vaccines. Here we demonstrated the presence of HPV-16 and -18 L1 genetic diversity (at the introduction of vaccination), which is important as baseline for the post vaccination surveillance.

In the past, several studies have addressed the intratypic variation of the major capsid protein from HPV-16 and/or HPV-18 [23–26]. Most of these studies were focused on small fragments of the L1 gene or were performed with limited sample numbers collected in Europe. Furthermore, in this regard no isolates collected in the Netherlands have been described previously. In this study we have evaluated the HPV-16 and HPV-18 L1 diversity based on 241 and 108 full-length HPV-16 and -18 L1 sequences, respectively, obtained from HPV isolates collected in the Netherlands around the introduction of HPV vaccination. The sequenced strains were representative for the total group in the PASSYON study, since the characteristics were non-significantly different from the total group, except for gender. A relatively low number of male sample were sequenced, but we expect no gender specific sequences. To our knowledge this is the largest study where the genetic diversity of the L1 gene in HPV-16 and -18 in HPV isolates collected in Europe is studied.

As HPV variants have been shown to differ in geographic origins [4] it was to be expected that European HPV-16 and -18 variants (lineage E/A) were identified most frequently in the Dutch isolates. Indeed, only a minority of our isolates concerned variant lineages, 7% and 14% for HPV-16 and -18 respectively, similar to the non-European variants from (sub)lineages B1, B2 (African1a and 1b), C (African 2a), D1 (North-American) and D2 and D3. Here we have found that all persons with a self-reported Surinam or Antillean origin of whom HPV-18 variant was analyzed carried a non-European HPV-18 variant. An earlier study had described that the distribution and persistence of HPV-16 and -18 variants may be related to the racial composition of individuals [27]. The present study supports this observation although the number of isolates from non-European variants was very small and the information on population group is based fully on self-reported data, the distribution of HPV-18 variants in the Netherlands seems to be ethnicity related. For HPV-16 variants this ethnicity-related distribution of variants in our Dutch isolates is not seen. It is not known if the ethnicity-associated variant distribution is caused by (long-term) mixing patterns in the population or if genetic factors which preferentially predispose persons are involved [27].

Our study identified known and new variants of HPV-16 and -18, with amino-acid variations in or close to epitopes. The new variants of HPV-16 and -18 were found both in European and non-European group of viral strains. Whether these changed L1 proteins are less susceptible to the vaccine induced immunity is unclear at this time. In general, human papillomaviruses mutate slowly [28] because they are double-stranded DNA viruses and use the proofreading DNA polymerase from their host. Nevertheless, nucleotide polymorphism can occur and become established in the population. Based on what is known about the evolution of the HPV genome, it is considered unlikely that HPV will show significant change in response to the human vaccination [29]. Accordingly, in our study population where HPV isolates collected around the introduction of HPV vaccination were studied, we found no evidence for selective pressure on the HPV-16 L1 gene (dN/dS ratio 0.176) or on the HPV-18 L1 gene (dN/dS ratio 0.172). Both ratios were less than one indicating that these sequences are under purifying selective pressure in the pre-vaccination period.

Some study limitations should be noted. First, we have sequenced a relative small number of HPV strains, especially strains sequenced from males and persons from the non-Dutch population group were low in number. Specific variants are not expected in males but have been seen in persons from non-Dutch population group. A second limitation is that we have selected swabs collected in 2009 and 2011 for studying the pre-vaccination genetic diversity. However, vaccination was introduced in the Netherlands in 2009 in a catch-up campaign in girls aged 14- to 16-years old before introduction in 12-year old girls in 2010. Therefore it is possible that samples collected in 2011 from 16–18 year old girls were from vaccinated girls. Since the number of strains collected in 2011 from 16 to 18 years girls is very low and the vaccination uptake at that time was about 50% we believe that no (or very little) dilution of vaccination effects have been introduced the pre-vaccination genetic diversity.

Whether in the future we will observe the emergence or expansion of (new) HPV-16- and or HPV-18 variants caused by the selective pressure induced by mass vaccination will remain unclear until investigated. Knowledge of the genetic diversity of HPV-16 and -18 at the start of vaccination (baseline), as is presented in this study is essential in order to understand the genetic variability of these proteins over time.

## Supporting Information

S1 Table Primers used for the molecular characterization of HPV-16 and HPV-18 L1 gene. (XLSX)

## References

[1] Burk RD, Harari A, Chen Z. Human papillomavirus genome variants. Virology. 2013;445(1–2):232–43. 10.10166/j.virol.2013.07.018 23998342 PMC3979972

[2] Munoz N, Bosch FX, de Sanjose S, Herrero R, Castellsague X, Shah KV, et al Epidemiologic classification of human papillomavirus types associated with cervical cancer. N Engl J Med. 2003;348(6):518–27. 10.1056/NEJMoa021641 . 12571259

[3] de Sanjose S, Quint WG, Alemany L, Geraets DT, Klaustermeier JE, Lloveras B, et al Human papillomavirus genotype attribution in invasive cervical cancer: a retrospective cross-sectional worldwide study. Lancet Oncol. 2010;11(11):1048–56. 10.1016/S1470-2045(10)70230-8 . 20952254

[4] Ho L, Chan SY, Burk RD, Das BC, Fujinaga K, Icenogle JP, et al The genetic drift of human papillomavirus type 16 is a means of reconstructing prehistoric viral spread and the movement of ancient human populations. Journal of virology. 1993;67(11):6413–23. 8411343 10.1128/jvi.67.11.6413-6423.1993 PMC238076

[5] Bernard HU, Calleja-Macias IE, Dunn ST. Genome variation of human papillomavirus types: phylogenetic and medical implications. International journal of cancer Journal international du cancer. 2006;118(5):1071–6. Epub 2005/12/07. 10.1002/ijc.21655 . 16331617

[6] Schiffman M, Wentzensen N. From human papillomavirus to cervical cancer. Obstetrics and gynecology. 2010;116(1):177–85. 10.1097/AOG.0b013e3181e4629f . 20567185

[7] Burk RD, Terai M, Gravitt PE, Brinton LA, Kurman RJ, Barnes WA, et al Distribution of human papillomavirus types 16 and 18 variants in squamous cell carcinomas and adenocarcinomas of the cervix. Cancer research. 2003;63(21):7215–20. . 14612516

[8] Cornet I, Gheit T, Clifford GM, Combes JD, Dalstein V, Franceschi S, et al Human papillomavirus type 16 E6 variants in France and risk of viral persistence. Infect Agent Cancer. 2013;8(1):4 10.1186/1750-9378-8-4 23343041 PMC3562255

[9] Villa LL, Sichero L, Rahal P, Caballero O, Ferenczy A, Rohan T, et al Molecular variants of human papillomavirus types 16 and 18 preferentially associated with cervical neoplasia. The Journal of general virology. 2000;81(Pt 12):2959–68. . 11086127 10.1099/0022-1317-81-12-2959

[10] Xi LF, Koutsky LA, Hildesheim A, Galloway DA, Wheeler CM, Winer RL, et al Risk for high-grade cervical intraepithelial neoplasia associated with variants of human papillomavirus types 16 and 18. Cancer epidemiology, biomarkers & prevention: a publication of the American Association for Cancer Research, cosponsored by the American Society of Preventive Oncology. 2007;16(1):4–10. 10.1158/1055-9965.EPI-06-0670 . 17220325

[11] Mollers M, Boot Hein J, Vriend Henrike J, King Audrey J, van den Broek Ingrid VF, van Bergen Jan EA, et al Prevalence, incidence and persistence of genital HPV infections in a large cohort of sexually active young women in the Netherlands. Vaccine. 2013;31(2):394–401. 10.1016/j.vaccine.2012.10.087 . 23146675

[12] Vriend HJ, Boot HJ, van der Sande MA, Medical Microbiological L, Municipal Health S. Type-specific human papillomavirus infections among young heterosexual male and female STI clinic attendees. Sex Transm Dis. 2012;39(1):72–8. 10.1097/OLQ.0b013e318235b3b0 . 22183851

[13] Vriend HJ, Bogaards JA, van der Klis FR, Scherpenisse M, Boot HJ, King AJ, et al Patterns of human papillomavirus DNA and antibody positivity in young males and females, suggesting a site-specific natural course of infection. PloS one. 2013;8(4):e60696 10.1371/journal.pone.0060696 23637760 PMC3634056

[14] Bishop B, Dasgupta J, Klein M, Garcea RL, Christensen ND, Zhao R, et al Crystal structures of four types of human papillomavirus L1 capsid proteins: understanding the specificity of neutralizing monoclonal antibodies. J Biol Chem. 2007;282(43):31803–11. 10.1074/jbc.M706380200 . 17804402

[15] Malagon T, Drolet M, Boily MC, Franco EL, Jit M, Brisson J, et al Cross-protective efficacy of two human papillomavirus vaccines: a systematic review and meta-analysis. Lancet Infect Dis. 2012;12(10):781–9. 10.1016/S1473-3099(12)70187-1 . 22920953

[16] Christensen ND, Dillner J, Eklund C, Carter JJ, Wipf GC, Reed CA, et al Surface conformational and linear epitopes on HPV-16 and HPV-18 L1 virus-like particles as defined by monoclonal antibodies. Virology. 1996;223(1):174–84. 10.1006/viro.1996.0466 . 8806551

[17] Varsani A, Williamson AL, Jaffer MA, Rybicki EP. A deletion and point mutation study of the human papillomavirus type 16 major capsid gene. Virus Res. 2006;122(1–2):154–63. 10.1016/j.virusres.2006.07.012 . 16938363

[18] Cornut G, Gagnon S, Hankins C, Money D, Pourreaux K, Franco EL, et al Polymorphism of the capsid L1 gene of human papillomavirus types 31, 33, and 35. Journal of medical virology. 2010;82(7):1168–78. 10.1002/jmv.21777 . 20513080

[19] Nei M, Gojobori T. Simple methods for estimating the numbers of synonymous and nonsynonymous nucleotide substitutions. Mol Biol Evol. 1986;3(5):418–26. . 3444411 10.1093/oxfordjournals.molbev.a040410

[20] Korber B. HIV Signature and Sequence variation analysis Computational Analysis of HIV Molecular Sequences, Chapter 4, Allen G Rodrigo and Gerald H Learn, eds Dordrecht, Netherlands: Kluwer Academic Publishers 2000:55–72.

[21] Pastrana DV, Vass WC, Lowy DR, Schiller JT. NHPV16 VLP vaccine induces human antibodies that neutralize divergent variants of HPV16. Virology. 2001;279(1):361–9. 10.1006/viro.2000.0702 . 11145917

[22] Roden RB, Armstrong A, Haderer P, Christensen ND, Hubbert NL, Lowy DR, et al Characterization of a human papillomavirus type 16 variant-dependent neutralizing epitope. Journal of virology. 1997;71(8):6247–52. 9223527 10.1128/jvi.71.8.6247-6252.1997 PMC191893

[23] Gagnon S, Hankins C, Money D, Pourreaux K, Canadian Women's HIVSG, Franco E, et al Polymorphism of the L1 capsid gene and persistence of human papillomavirus type 52 infection in women at high risk or infected by HIV. Journal of acquired immune deficiency syndromes. 2007;44(1):61–5. 10.1097/01.qai.0000247226.45375.01 . 17075388

[24] Stewart AC, Eriksson AM, Manos MM, Munoz N, Bosch FX, Peto J, et al Intratype variation in 12 human papillomavirus types: a worldwide perspective. Journal of virology. 1996;70(5):3127–36. 8627792 10.1128/jvi.70.5.3127-3136.1996 PMC190175

[25] Frati E, Bianchi S, Colzani D, Zappa A, Orlando G, Tanzi E. Genetic variability in the major capsid L1 protein of human papillomavirus type 16 (HPV-16) and 18 (HPV-18). Infection, genetics and evolution: journal of molecular epidemiology and evolutionary genetics in infectious diseases. 2011;11(8):2119–24. 10.1016/j.meegid.2011.06.014 . 21729769

[26] Yue Y, Yang H, Wu K, Yang L, Chen J, Huang X, et al Genetic variability in L1 and L2 genes of HPV-16 and HPV-58 in Southwest China. PloS one. 2013;8(1):e55204 10.1371/journal.pone.0055204 23372836 PMC3555822

[27] Xi LF, Kiviat NB, Hildesheim A, Galloway DA, Wheeler CM, Ho J, et al Human papillomavirus type 16 and 18 variants: race-related distribution and persistence. Journal of the National Cancer Institute. 2006;98(15):1045–52. 10.1093/jnci/djj297 . 16882941

[28] Rector A, Lemey P, Tachezy R, Mostmans S, Ghim SJ, Van Doorslaer K, et al Ancient papillomavirus-host co-speciation in Felidae. Genome Biol. 2007;8(4):R57. Epub 2007/04/14. doi: gb-2007-8-4-r57 [pii] 10.1186/gb-2007-8-4-r57 17430578 PMC1896010

[29] Chen Z, Schiffman M, Herrero R, DeSalle R, Anastos K, Segondy M, et al Evolution and taxonomic classification of alphapapillomavirus 7 complete genomes: HPV18, HPV39, HPV45, HPV59, HPV68 and HPV70. PloS one. 2013;8(8):e72565 10.1371/journal.pone.0072565 23977318 PMC3745470

