# Correction: Genetic Diversity in the Major Capsid L1 Protein of HPV-16 and HPV-18 in the Netherlands

**DOI:** 10.1371/journal.pone.0154977

**Published:** 2016-04-28

**Authors:** Audrey J. King, Jan A. Sonsma, Henrike J. Vriend, Marianne A. B. van der Sande, Mariet C. Feltkamp, Hein J. Boot, Marion P. G. Koopmans, on behalf of Medical Microbiological Laboratories and Municipal Health Services

In the Data Availability statement and the Materials and Methods section, the GenBank accession number KU70477 appears incorrectly. The correct accession number should be KU707477.
